# How pride works

**DOI:** 10.1017/ehs.2021.6

**Published:** 2021-02-01

**Authors:** Daniel Sznycer, Adam Scott Cohen

**Affiliations:** 1 Department of Psychology, University of Montreal, Montreal, QC, Canada; 2 Universidad Francisco Marroquín, Guatemala City, Guatemala; 3 University of Hawai'i at Manoa, Honolulu, HI, USA

**Keywords:** emotion, motivation, valuation, evolutionary psychology, culture

## Abstract

The emotion of pride appears to be a neurocognitive guidance system to capitalize on opportunities to become more highly valued and respected by others. Whereas the inputs and the outputs of pride are relatively well understood, little is known about how the pride system matches inputs to outputs. How does pride work? Here we evaluate the hypothesis that pride magnitude matches the various outputs it controls to the present activating conditions – the precise degree to which others would value the focal individual if the individual achieved a particular achievement. Operating in this manner would allow the pride system to balance the competing demands of effectiveness and economy, to avoid the dual costs of under-deploying and over-deploying its outputs. To test this hypothesis, we measured people's responses regarding each of 25 socially valued traits. We observed the predicted magnitude matchings. The intensities of the pride feeling and of various motivations of pride (communicating the achievement, demanding better treatment, investing in the valued trait and pursuing new challenges) vary in proportion: (a) to one another; and (b) to the degree to which audiences value each achievement. These patterns of magnitude matching were observed both within and between the USA and India. These findings suggest that pride works cost-effectively, promoting the pursuit of achievements and facilitating the gains from others’ valuations that make those achievements worth pursuing.

## Graphical abstract

**Figure:**
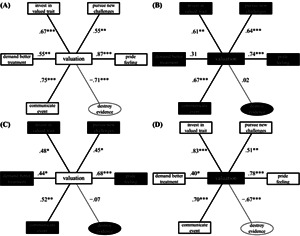

**Social media summary:** Pride works by promoting achievements and facilitating the valuation from others that make achievements worth pursuing.

## Introduction

Being valued by other people is a key resource for humans (Baumeister & Leary, 1995). When other people value you, they are disposed to attend to you, to defer to you, to help you when you are in need and to forego opportunities to benefit at your expense (Vaughn & Waters, 1981; DeScioli & Kurzban, 2009; Delton, 2010; Von Rueden et al., 2010; Sznycer et al., 2019). And when other people do not value you, they are not so disposed.

When other people (an audience) detect new information about a target individual revealing that their valuations of the target individual are outdated, others appear to recalibrate how much they value the target, up or down, with correspondingly positive or negative effects on the target's welfare and fitness (Tooby et al., 2008; Sznycer, 2019). This may have selected, at the individual's end, for motivational systems to pursue socially valued courses of action and to cultivate socially valued characteristics, to refrain from pursuing socially disvalued courses of action (or, when those are personally profitable, to pursue them when circumstances are auspicious), to advertise reputation-enhancing information and to conceal reputation-damaging information (Leary & Kowalski, 1990).

These considerations are relevant to the emotion of pride. Recent theorizing suggests that pride functions to incentivize fellow community members to attach more weight to the welfare of the individual. An emotion realizing this adaptive function is expected to (a) motivate the pursuit of actions or the cultivation of characteristics that are socially valued (or feared), (b) motivate the advertisement of socially valued actions and characteristics and (c) motivate the individual to profit from the resulting enhanced valuation from others (e.g. by demanding better treatment from others, by pursuing new challenges previously beyond reach). This constitutes an advertisement–recalibration theory of pride (Sznycer et al., 2017, 2018b; Sznycer, 2019; Durkee et al., 2019; Cohen et al., 2020; see also: Tracy et al., 2010; Tracy & Matsumoto, 2008; Fessler, 1999; Weisfeld & Dillon, 2012).

To illustrate how pride may operate, consider the following hypothetical situation: Anne decides to cultivate weather-forecasting skills because she estimates that the benefits she will receive from others if she has those skills will more than offset the costs of acquiring those skills. Eventually, Anne learns how to forecast the weather. Because of this, Anne's neighbours now can better prepare for severe weather events. Anne advertises her skills, and her neighbours correspondingly increase how much they value Anne; now they benefit her at a higher rate (e.g. they help her more) and impose costs on her at a lower rate (e.g. they benefit at her expense less).

The existing evidence is consistent with this theory. Pride appears to be a human-universal emotion, present in all of the world's cultures (Brown, 1991) and appearing reliably and early in development (Lewis et al., 1992; Stipek, 1995). On the input side, pride is elicited by actions and personal characteristics indicating enhanced capacity to confer benefits or impose costs on others (Lewis et al., 1992; Tracy & Matsumoto, 2008; Weisfeld & Beresford, 1982; Schniter et al., 2020). On the output side, pride produces a highly pleasant feeling (Mauro et al., 1992) which can reinforce behaviour leading to achievements (Gilchrist et al., 2018; Riskind, 1984). In addition, pride produces a full-body display featuring expanded posture and gaze directed at the audience (Fessler, 1999; Tracy & Matsumoto, 2008; Weisfeld & Dillon, 2012). Audiences interpret the pride display as an indication of the displayer's achievements or formidability (Fessler, 1999; Weisfeld & Dillon, 2012; Shariff & Tracy, 2009; Tiedens et al., 2000). Further, the pride display is recognized across cultures (Tracy & Robins, 2008).

## Balancing effectiveness against economy

To make decisions adaptively, the human mind–brain needs to predict and integrate two types of payoffs: (a) the direct payoff of a candidate action (e.g. the personal cost of foraging for a food item added to the personal benefit of acquiring that food item); and (b) the social valuation payoff (e.g. the increased valuation from audiences that accrues when the individual demonstrates her foraging skills). It has been argued that the pride that people feel prospectively, in anticipation of taking a candidate action, is an internal signal of the estimated social valuation payoff – a signal that entrains motivational, decisional and physiological systems which, jointly, can lead to the pursuit of socially valued courses of action, the advertisement of achievements, and the cashing in on others’ higher valuation of the self (Sznycer et al., 2017).

According to the advertisement–recalibration theory, (a) the pride system predicts, for each candidate course of action considered, the degree to which the audience will value the individual if the individual achieves the corresponding achievement (and the audience learns about the achievement) and (b) the pride system mobilizes its outputs incrementally, with an intensity that is proportional to those predictions on an event-by-event basis. This allows the pride system to guide decisions adaptively: a candidate course of action will be pursued if its combined direct and social payoff is estimated to be positive, and higher than those of the alternatives considered.

Pride is expected to embody the Goldilocks principle and be mobilized to a degree that is just right. The under-mobilization of pride relative to the actual magnitude of audience valuation for a given achievement would lead to maladaptive decisions where, e.g., the relevant course of action is under-pursued. Conversely, the over-mobilization of pride would lead to over-pursuing valued actions, over-advertising achievements and over-claiming valuation and respect – something which audiences resist and devalue (Schlenker & Leary, 1982). However, these dual errors are avoided if pride can accurately predict how much valuation audiences will confer on the individual for a candidate action and mobilize in proportion to that prediction. Note that, whereas pride is expected to be on average well calibrated to the valuations of audiences, it may occasionally be dysregulated in either direction. There are individual-level differences in pride (Alessandri & Lewis, 1996; Belsky et al., 1997), and these are likely to be underlain in part by genetic or developmental noise and in part by adaptive calibration (e.g. if individuals vary in their capacity to produce benefits and thus in their capacity to gain valuation from others; see Tooby & Cosmides, 1990a).

Recent research has tested the predicted match to audience valuation regarding one output of the pride system – the anticipatory feeling of pride. As predicted, the intensity of the anticipatory feeling of pride with respect to a candidate act or personal characteristic closely tracks the degree to which audiences positively value those individuals who take those acts or possess those characteristics. This is so in mass societies (Sznycer et al., 2017; Sznycer & Lukaszewski, 2019; Durkee et al., 2019; Cohen et al., 2020) and in traditional small-scale societies (Sznycer et al., 2018b).

## Do multiple pride responses track the valuations of audiences?

Much is known about the inputs and the outputs of pride. In contrast, the internal logic of this emotion has been relatively unexplored. How does pride match inputs to outputs? Here we evaluate the broader hypothesis that multiple outputs available to the pride system – and not only the pride feeling – match in intensity the degree to which audiences value individuals based on the individuals' actions and characteristics on an event-by-event basis. This is expected if the Goldilocks principle superintends the various functional subcomponents of pride. If so, then multiple responses of pride will all cohere in direction and intensity with one another and with the valuations conferred by audiences.

Note, however, that whereas multiple pride responses are expected to cohere in the general case, responses may fail to cohere when tactical considerations render coherence a suboptimal bet (Kyl-Heku & Buss, 1996; Lukaszewski et al., 2020; Wood et al., 2015). For instance, an individual may pursue a socially valued course of action but fail to advertise it if the achievement fails to materialize, or if an audience is not co-present when the achievement occurs (Fridlund, 1991). This type of context effect or adaptive contingency – not evaluated or theorized comprehensively in the present paper – may explain why response coherence has been observed inconsistently in previous emotion research (for overview, see Hollenstein & Lanteigne, 2014).

Inconsistencies in response coherence in emotion may be due to the functional type of context effect noted above (Reisenzein, 2000; Sznycer & Cohen, under review) – although inconsistencies in response coherence may also be due to noise, to methodological complexities (Hollenstein & Lanteigne, 2014) and, at the limit, to emotion programmes lacking biological reality (Barrett, 2006a). Indeed, some researchers have interpreted observations of inconsistent response coherence, and of limited response specificity (Barrett et al., 2019; Siegel et al., 2018), to mean that emotion episodes emerge from the interaction of neurocognitive systems that are not themselves emotion systems – that emotions are not natural kinds (Barrett, 2006a; Barrett & Russell, 2015).

According to the alternative theory of constructed emotion (Barrett & Russell, 2015), emotion episodes emerge not from the operation of emotion programmes with orchestrating functions but from the focal individual's concept-assisted categorization of her internal signals of valence (feelings of pleasure or displeasure) and arousal (the state of being excited vs. lethargic), jointly termed ‘core affect’ (Barrett, 2017). Under this alternative theory, the machinery that generates core affect, the concepts that parse core affect and the behaviours and physiology deployed in emotional episodes (e.g. the fight-or-flight response) *are* biologically real. However, orchestrating emotion systems as such are not biologically real. Further, emotion episodes are highly culturally idiosyncratic, because the concepts that parse affect can vary highly across cultures (Barrett, 2006b; Jackson et al., 2019). Note that if pride is a constructed emotion, then response coherence in pride episodes, if any, will arise to the extent that valence, arousal, or concepts are similar across episodes, individuals, or cultures – but not from an evolved pride orchestrator (which, to reiterate, has no existence). We return to this alternative account of pride below.

The aim of the present study is to establish whether coherence in pride responses can arise. Therefore, the present study is designed to minimize the coherence-reducing context effects mentioned above. We give study participants skeletal information about socially valued actions and personal characteristics but otherwise provide little or no information about situational variables that might moderate the operation of pride and decrease its response coherence (e.g. the presence or absence of an audience, the characteristics of the audience, the way the audience actually responds).

The present study evaluates anticipatory responses of pride. A key function of pride (Sznycer et al., 2017; Van Der Schalk et al., 2012) and other emotions (Bechara et al., 2000) is to evaluate alternative future courses of action in order to guide action. Thus, it is expected that the anticipated intensities of various responses of pride will cohere with one another and with the magnitude of positive evaluations expressed by the audience for the relevant acts and personal characteristics.

## Predictions

The following predictions are adapted from research on the outputs of shame by Sznycer and Cohen (under review). If the pride system is a standard feature of the human mind–brain, and if pride has authority over responses *a*, *b*, and *c*, the following will come to pass. First, in some situations, responses *a*, *b*, and *c* will be mobilized in proportion to one other and in the direction or manner that is mandated by pride. This is a prediction about internal response coherence.

Previous emotion research on response coherence has focused on *internal* response coherence. The advertisement–recalibration theory can explain why internal coherence occurs – because the various responses under emotion control may have all been selected to balance the competing demands of effectiveness and economy. However, in addition, the advertisement–recalibration theory can generate novel predictions. Next, we outline some of these.

In some situations – second prediction – responses *a*, *b*, and *c* will be mobilized in proportion to the magnitude of the opportunity that pride functions to exploit – the opportunity to become more highly valued by others and capture the attendant benefits. This is expected, as argued above, because the various responses under pride control can better perform their respective functions if they are mobilized just right – neither insufficiently nor excessively. This is a prediction about external coherence between responses or outputs of the pride system on the one hand and the target domain of the pride system – the social valuation of audiences – on the other hand.

Third, in some situations, internal and external coherence will be observed within cultures worldwide.

Fourth, coherence may be observed between cultures. For example, the more a personal characteristic is considered admirable by an audience in culture 1, the more individuals who possess that characteristic may display pride response *a* (and *b* and *c*) in culture 2. Pride is tuned specifically to how various actions and personal characteristics are appraised in the individual's own *local* social ecology (Sznycer et al., 2017). This local tuning seems to be a design feature, because the evaluative opportunities that pride needs to take advantage of are a function of the particular actions and characteristics that *one's fellow group members* find attractive, helpful, instrumental or praiseworthy. Nevertheless, if there are cross-cultural regularities in how people value other people, then some actions and personal characteristics that are viewed as attractive, virtuous, odd, anger-provoking or immoral may be similar across cultures. Indeed, there is evidence of cross-cultural commonalities in the things that people value or disvalue in other people (Brown, 1991; Curry et al., 2019; Hanel et al., 2018; Durkee et al., 2019; Shackelford et al., 2005; Sznycer et al., 2016a, b, 2018a; Sznycer & Patrick, 2020; Sell et al., 2017; Petersen et al., 2012). Thus, response coherence between cultures may be expected sometimes.

Finally, internal, external and cross-cultural coherence will arise functionally, through the operation of the pride system. Here, we consider the possibility that coherence, if observed, is a concomitant of other causes – something which has been evaluated infrequently despite its importance, as Barrett and others have argued (e.g. Barrett, 2006a, 2012). As noted above, according to the alternative theory of constructed emotion, emotion episodes emerge when the individual uses emotion concepts to categorize her own internal signals of valence and arousal. Therefore, under this alternative theory, cross-situational, cross-individual and cross-cultural coherence in pride, if any, will stem from: (a) similarities in the relevant concepts and acts of categorization; (b) similarities in valence; or (c) similarities in arousal – but emphatically not from the action of an evolved pride orchestrator that is part of human nature (which lacks biological reality under this alternative theory). Here we consider the possibility that response coherence in pride, if observed, is *not* imparted by design, by a pride orchestrator that functions to promote valuation from others (as the advertisement–recalibration theory holds), but is instead a concomitant of, for instance, arousal (as the alternative theory of constructed emotion might predict). Pride episodes are arousing (Nelson & Russell, 2014). If the state of arousal in pride episodes makes people want to act, and if a valuation-promoting pride orchestrator does not in fact exist (as the alternative theory of constructed emotion holds), then more intense (and arousing) feelings of pride may cohere with more intense motivations to take agentic actions (vs. lethargy), including communicating the event, demanding better treatment, investing in the valued trait or course of action, pursuing new challenges, *and destroying evidence about the individual's own achievement* – actions that are similarly arousing (vs. lethargic) but dissimilar regarding their potential to get others to value the self. In contrast, if a valuation-promoting pride orchestrator does in fact exist, and if pride coordinates various responses in Goldilocks fashion in order to incentivize others to increase how much they value the self, then responses that promote positive evaluations from others (pride feeling, communicate event, demand better treatment, invest in valued trait, and pursue new challenges) will cohere with one other but will not cohere with responses that impede or diminish positive evaluations from others such as destroying evidence about one's own achievements. That is, here we evaluate whether coherence, if observed, can be explained by an alternative that features a low-level affective property of pride episodes (high arousal) but otherwise lacks the adaptive functionality of promoting valuation in others (because this is not an explanatory element of the theory of constructed emotion). We intend this as an initial test against the alternative theory of constructed emotion.

In sum, it is predicted that response coherence in pride can, in some situations, be observed at multiple levels: internally, externally and cross-culturally. Moreover, response coherence will arise functionally, through the operation of pride, rather than through lower-level variables such as arousal.

## The present study

We measure, for each of 25 socially valued actions and personal characteristics, the degree to which participants, from the perspective of an audience, would socially value a target individual if that individual took those actions or possessed those characteristics. We also measure the degree to which each of those 25 actions and characteristics, if true of participants, would elicit in participants five pride responses: felt pride, as well as the motivations to communicate the event, demand better treatment, invest in the valued trait and pursue new challenges. Finally, we measure, for each of the 25 actions and characteristics, a motivation that is not predicted to increase with the intensity of pride: the motivation to destroy evidence about the achievement. Importantly, the 25 actions and characteristics are specified in skeletal form, with little or no information about various situational factors that might decrease response coherence.

By correlating the intensities of the various pride responses, we can determine whether these responses cohere with one another and with the intensity of audience valuation in the manner that is predicted by the hypothesis that pride is a valuation-promoting emotion. We conduct this study in two populations with disparate cultures, the USA and India, to establish whether the predicted patterns of response coherence are observed within and across cultures.

## Method

Procedure, stimuli, sample sizes, exclusion criteria, predictions and analyses were preregistered before data collection began (https://aspredicted.org/4k5wq.pdf). The dataset is available in the OSF repository (https://osf.io/tr8fe/).

### Participants and procedure

Standard power analyses to determine sample size of participants were not conducted because the correlations are computed over the sample of scenarios (fixed in quantity), not over participants. However, pilot data suggested that 25 participants per condition per country yield adequate power. This number was supplemented to compensate for likely exclusions owing to participant inattention. We assumed 30% of data exclusions owing to inattention. Thus, we set the total number of participants to be recruited per country to 245–35 participants per condition.

We collected data with Amazon Mechanical Turk from 245 participants (144 females) in the USA and 241 participants (78 females) in India. As per the preregistration protocol, participants were excluded from analyses if they failed to pass an attention check. Two American participants and 74 Indian participants were excluded from analyses owing to inattention, leaving an effective sample of 243 American participants (143 females; mean age 39 years, SD = 12) and 167 Indian participants (55 females; mean age 29 years, SD = 7).

The stimuli consist of 25 brief hypothetical scenarios, developed by Sznycer et al. (2017), in which someone's acts, traits or circumstances might lead them to be viewed positively by others. The scenarios were designed to elicit reactions in a wide variety of evolutionarily relevant domains, such as social exchange, friendship, aggressive contests, mating, parenting and leadership, and were phrased at a relatively high level of abstraction to make it likely that their meanings would be understood across cultures.

Participants were randomly assigned to one of seven conditions in a between-subjects design: (a) a *valuation* condition, and (b–g) six conditions relevant to pride – (b) *pride feeling*, (c) *communicate event*, (d) *demand better treatment*, (e) *invest in valued trait*, (f) *pursue new challenges* and (g) *destroy evidence*. In all seven conditions participants rated the same basic set of 25 scenarios. The main difference across conditions – the experimental manipulation – was a prompt, displayed immediately before the scenarios, instructing participants to interpret the scenarios in a way that would elicit either valuation of a target individual or one of the six pride-relevant responses.

In the valuation condition, the prompt asked participants to imagine that the acts, traits or circumstances described in the 25 scenarios (e.g. ‘She is trustworthy’, ‘She has many unique skills’, ‘She finished first in a marathon’, ‘She is ambitious’) are true of a target individual: an individual other than the participant who is of the same sex and age as the participant. Then, participants were asked to indicate, for each scenario, ‘how positively you would view this person if those things were true of that person’, with scales ranging from 1 (‘I'd view her not positively at all if this were true of her’) to 7 (‘I'd view her very positively if this were true of her’). These ratings provide event-specific measures of the degree to which members of a given population would socially value the individual described in the scenarios.

In the six other conditions (pride feeling, communicate event, demand better treatment, invest in valued trait, pursue new challenges and destroy evidence), the prompts asked participants to imagine that the acts, traits or circumstances described in the 25 scenarios are true of the participant herself (e.g. ‘You are trustworthy’, ‘You have many unique skills’, ‘You finished first in a marathon’, ‘You are ambitious’), and to indicate the degree to which they would experience feelings or motivations relevant to pride on scales ranging from 1 (not at all …) to 7 (a lot … / very much …). The prompts asked participants to indicate the following. In the pride feeling condition: how much pride they would feel if the things described in the scenarios were true of them. In the communicate event condition: how willing they would be to communicate to others that those things are true of them. In the demand better treatment condition: how willing they would be to demand others to treat them better because of those things. In the invest in valued trait condition: how willing they would be to invest resources (time, effort, etc.) so that those things continued to be true of them. In the pursue new challenges condition: how much, because of those things, they would be motivated to pursue new life challenges. In the destroy evidence condition: how willing they would be to destroy evidence or clues that might tell others that those things are true of them.

The scenarios were presented in randomized order within conditions. The stimuli were presented in English in the USA and India. Full text of the condition prompts and scenarios used in the USA and India is provided in the Online Appendix, Tables S1–S3.

## Results

### Within-country results

First, we report the results for each country. Descriptive statistics are provided in Tables S2 and S3.

*Do participants within countries agree on how positively they would view the target individual in each of these scenarios?* Yes. To measure agreement among raters about the relative extent to which they would value a target individual if 25 acts and traits were true of that individual we computed intra-class correlations (ICC) in each country. Raters agreed about the relative extent to which they would value the target individual: USA, ICC (2,34) = 0.96; India, ICC (2,25) = 0.79, *P* < 0.001.

*Do participants within countries agree on the degree to which they would experience one of the five pride responses if the acts and traits described in the scenarios were true of them?* In the USA there was widespread agreement about the relative intensities of pride responses that the 25 situations would elicit: pride feeling, ICC (2,36) = 0.94; communicate event, ICC (2,36) = 0.93; demand better treatment, ICC (2,35) = 0.80; invest in valued trait, ICC (2,35) = 0.95; pursue new challenges, ICC (2,33) = 0.90, *P* < 0.001. All of the intra-class correlations in the USA remain significant after applying, as per the preregistration, a false discovery rate (FDR) correction of *P* < 0.05 (Benjamini & Hochberg, 1995). In India, there was agreement about the relative intensity of pride feeling, ICC (2,26) = 0.60, and invest in valued trait, ICC (2,21) = 0.52 (*P* < 0.01), but there was no agreement for communicate event, ICC (2,26) = 0.19, demand better treatment, ICC (2,19) = −0.02, or pursue new challenges, ICC (2,23) = 0.34 (*P* ≥ 0.059). In India, the intra-class correlations of valuation, pride feeling and invest in valued trait remain significant at FDR *P* < 0.05; all of the other intra-class correlations in India (communicate event, demand better treatment, pursue new challenges) are not significant at FDR *P* < 0.05.

**Figure 1.**
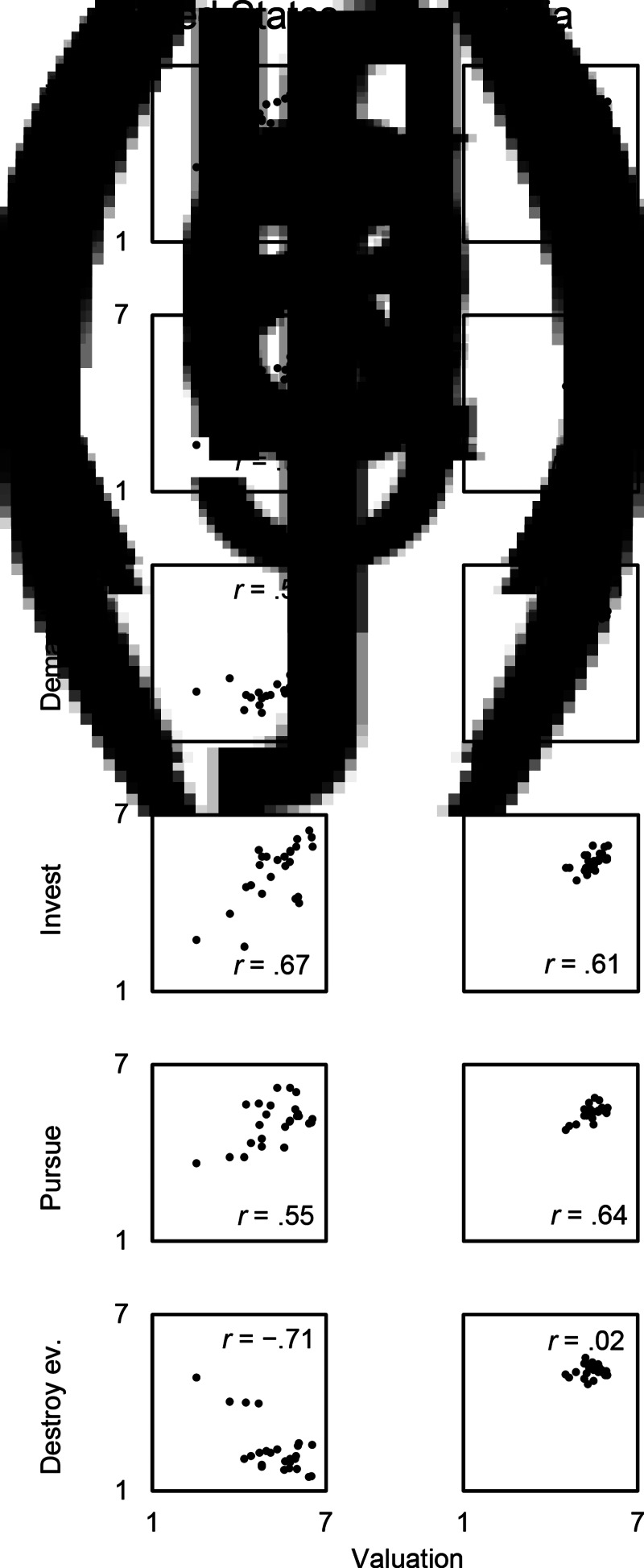
Scatter plots: intensities of pride-relevant outputs as a function of valuation, by country. Note: each point represents the mean valuation rating and mean output rating of one scenario. Ratings of valuation, pride feeling, communicate event, demand better treatment, invest in valued trait, pursue new challenges and destroy evidence were given by different participants. Number on which the correlations are based = number of scenarios = 25. USA data, panels a–f; India data, panels g–l.

**Figure 2.**
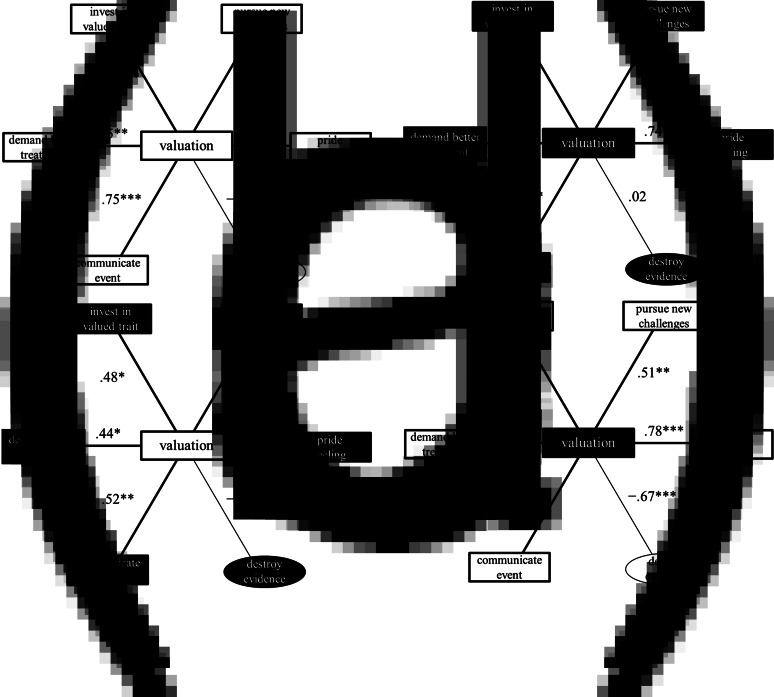
Correlations between ratings of valuation and ratings of pride feeling, communicate event, demand better treatment, invest in valued trait, pursue new challenges and destroy evidence, within and between countries. Note: (a) USA correlations (white shapes); (b) India correlations (black shapes); (c) Correlations between valuation in the USA and pride-relevant outputs in India; (d) correlations between valuation in India and pride-relevant outputs in the USA. Number on which the correlations are based = number of scenarios = 25. Ratings of valuation, pride feeling, communicate event, demand better treatment, invest in valued trait, pursue new challenges and destroy evidence were given by different participants. ***P < 0.001; **P < 0.01; *P < 0.05.

*Does the intensity of audience valuation correlate positively with the intensities of the five pride responses?* In general, yes. The intensity of social valuation that participants express (as audiences) if 25 socially valued acts and traits are true of someone else correlates positively with the intensities of pride feeling, communicate event, demand better treatment, invest in valued trait and pursue new challenges if those 25 positive acts and traits were true of the participants themselves. For each of the 25 scenarios, we calculated the mean ratings of each of the five pride responses provided by participants in the pride-relevant conditions and the mean valuation ratings provided by participants in the valuation condition. We computed Pearson correlation coefficients. In the USA, ratings of valuation positively correlated with the ratings of the five pride responses: pride feeling, communicate event, demand better treatment, invest in valued trait and pursue new challenges (*r* = 0.55–0.87, *p* = 10^−7^ to 0.005). In India, ratings of valuation positively correlated with the ratings of four pride responses: pride feeling, communicate event, invest in valued trait and pursue new challenges (*r* = 0.61–0.74, *P* = 0.00002–0.001). The correlation between ratings of valuation and ratings of demand better treatment was positive but not significant in India (*r* = 0.31, *P* = 0.13; Figs 1 and 2a, b, and Table S4). Recall that the ratings of valuation, pride feeling, communicate event, demand better treatment, invest in valued trait and pursue new challenges originated from different participants. Consequently, these correlations cannot be attributed to participants matching their valuation ratings to their pride response ratings.

We computed Bayes factors (BFs) to quantify the odds that the data favour each alternative hypothesis relative to their corresponding null hypotheses. The alternative hypothesis, which states that audience valuation would correlate with the five pride responses, was tested against the null hypothesis, which states that they would not correlate. Bayesian correlation analyses used default priors (stretched *β* prior width = 1; JASP 0.10.2). In the USA, each alternative hypothesis regarding the five pride responses (audience valuation correlates with the intensities of the five pride responses) was more likely than the null (all BFs_10_ > 10). In India, the alternative hypothesis was more likely than the null for four of the five pride responses (pride feeling, BF_10_ = 1.31 × 10^3^; communicate event, BF_10_ = 133; invest in valued trait, BF_10_ = 35.6; pursue new challenges, BF_10_ = 73.3), the exception was demand better treatment (BF_10_ = 0.74).

*Does the intensity of audience valuation fail to correlate positively with participants’ willingness to destroy evidence that might tell others that they (the participants) have socially valuable traits?* Yes. The correlation between ratings of valuation and ratings of destroy evidence was negative in the USA (*r* = −0.71, *P* = 0.00007) and close to zero in India (*r* = 0.02, *P* = 0.93). The alternative hypothesis (ratings of valuation and ratings of destroy evidence were correlated) was more likely than the null in the USA only (in the USA, BF_10_ = 439; in India, BF_10_ = 0.24).

*Do the intensities of the five pride responses correlate positively with one another?* In general, yes. In the USA, ratings of pride feeling, communicate event, demand better treatment, invest in valued trait and pursue new challenges are positively correlated with one other, with a mean *r* = 0.50 (SD = 0.18; minimum *r* = 0.20; maximum *r* = 0.82; number of *r* values = 10), *P* values = 10^−6^ to 0.34; 7 of these 10 correlations are significant at FDR *P* < 0.05 (Table S4). The alternative hypotheses in the USA were more likely than the null for 6 of 10 pairs of responses (pride feeling–communicate event, BF_10_ = 134; pride feeling–demand better treatment, BF_10_ = 3.18; pride feeling–invest in valued trait, BF_10_ = 2.72 × 10^4^; pride feeling–pursue new challenges, BF_10_ = 121; communicate event–invest in valued trait, BF_10_ = 7.91; invest in valued trait–pursue new challenges, BF_10_ = 8.20) and indeterminate for 4 of 10 pairs of responses (communicate event–demand better treatment, BF_10_ = 2.93; communicate event–pursue new challenges, BF_10_ = 0.38; demand better treatment–invest in valued trait, BF_10_ = 0.90; demand better treatment–pursue new challenges, BF_10_ = 1.25). In India, ratings of pride feeling, communicate event, demand better treatment, invest in valued trait, and pursue new challenges are positively correlated with one other, with a mean *r* = 0.41 (SD = 0.17; minimum *r* = 0.20; maximum *r* = 0.69; number of *r* values = 10), *P* values = 0.0002–0.35; 4 of these 10 correlations are significant at FDR *P* < 0.05 (Table S4). From a Bayesian perspective, the alternative hypothesis in India was more likely than the null for 4 of 10 pairs of responses (pride feeling–communicate event, BF_10_ = 8.06; pride feeling–pursue new challenges, BF_10_ = 209; communicate event–invest in valued trait, BF_10_ = 12.2; communicate event–pursue new challenges, BF_10_ = 24.6) and indeterminate for 6 of 10 pairs of responses (pride feeling–demand better treatment, BF_10_ = 0.38; pride feeling–invest in valued trait, BF_10_ = 1.13; communicate event–demand better treatment, BF_10_ = 1.99; demand better treatment–invest in valued trait, BF_10_ = 0.57; demand better treatment–pursue new challenges, BF_10_ = 0.37; invest in valued trait–pursue new challenges, BF_10_ = 0.66).

### Between-country results

To test for between-country agreement in valuation, in pride responses and in the valuation–pride-response links, we computed the extent to which the mean ratings of valuation and the mean ratings of pride responses are correlated across countries.

*Valuation: do American and Indian participants agree on how positively they would view the target individual in each of these scenarios?* Yes. There was between-country agreement on the degree to which a given socially valued act or trait would elicit valuation: *r* = 0.82, *P* = 10^−6^. The more American participants valued a target individual if the individual took an act or possessed a trait, the more Indian participants valued a target individual if the individual took that act or possessed that trait. The alternative hypothesis (valuation in USA and India correlated) was more likely than the null (BF_10_ = 3.77 × 10^4^).

*Pride responses: do American and Indian participants agree on the degree to which they would experience pride feelings and pride-relevant motivations?* Yes. American and Indian participants agreed about the relative extent to which a socially valued act or trait would elicit the feeling of pride (*r* = 0.76, *P* = 0.00001), as well as the motivations to communicate the event (*r* = 0.45, *P* = 0.03), demand better treatment (*r* = 0.55, *P* = 0.004), invest in the valued trait (*r* = 0.59, *P* = 0.002) and pursue new challenges (*r* = 0.57, *P* = 0.003). Further, in 11 of 20 cases a pride response in one country correlated positively and significantly with a different pride response in the other country (e.g. communicate event in the USA vs. demand better treatment in India; mean *r* = 0.40 (SD = 0.19; minimum *r* = 0.02; maximum *r* = 0.74; number of *r* values = 20), *P* values = 0.00002–0.91; Table S4). The alternative hypotheses that ratings of a given pride response would correlate across the two countries were more likely than the null for four of five pride responses (pride feeling, BF_10_ = 2.51 × 10^3^; demand better treatment, BF_10_ = 11.2; invest in valued trait, BF_10_ = 24.6; pursue new challenges, BF_10_ = 17.4) and indeterminate for one of five pride responses (communicate event, BF_10_ = 2.66). Among the 20 correlations between a pride response and a different pride response in the other country, the evidence favoured the alternative hypothesis of a correlation relative to the null for eight pairs (BFs_10_ between 7.13 and 1.18 × 10^3^), was indeterminate for nine pairs (BFs_10_ between 0.39 and 2.56) and favoured the null relative to the alternative for three pairs (BFs_10_ between 0.25 and 0.329).

*Does the intensity of valuation in one country correlate positively with the intensities of pride responses in the other country?* In general, yes. American participants’ ratings of valuation correlated positively with Indian participants’ ratings of pride feeling, communicate event, demand better treatment, invest in valued trait and pursue new challenges; mean *r* = 0.51 (SD = 0.10; minimum *r* = 0.44; maximum *r* = 0.68; number of *r* values = 5); *P* values = 0.0002–0.03. Likewise, Indian participants’ ratings of valuation correlated positively with American participants’ ratings of pride feeling, communicate event, demand better treatment, invest in valued trait and pursue new challenges; mean *r* = 0.65 (SD = 0.18; minimum *r* = 0.40; maximum *r* = 0.83; number of *r* values = 5), *P* values = 10^−6^ to 0.047 (Fig. 2c, d, Table S4). To put some of this more vividly, Indians’ willingness to invest in socially valued actions and traits is positively associated with Americans’ valuations of those actions and traits, and Americans’ willingness to communicate actions and traits to others is positively associated with Indians’ feelings of pride about those actions and traits. All of the cross-country correlations (valuation vs. valuation; pride response vs. same pride response; pride response vs. different pride response) that are significant at *P* < 0.05 also remain significant at FDR *P* < 0.05, except for three correlations: India invest in valued trait vs. US communicate event; India invest in valued trait vs. US demand better treatment; and India valuation vs. US demand better treatment. The alternative hypothesis that the intensity of audience valuation in the USA correlates with the intensities of pride responses in India was more likely than the null of no correlation for three of the five pride responses (pride feeling, BF_10_ = 193; communicate event, BF_10_ = 7.41; invest in valued trait, BF_10_ = 4.03) and indeterminate for two of five pride responses (demand better treatment, BF_10_ = 2.37; pursue new challenges, BF_10_ = 2.79). The alternative hypothesis that the intensity of audience valuation in India correlates with the intensities of pride responses in the USA was more likely than the null of no correlation for four of five pride responses (pride feeling, BF_10_ = 6.25 × 10^3^; communicate event, BF_10_ = 297; invest in valued trait, BF_10_ = 6.51 × 10^4^; pursue new challenges, BF_10_ = 6.11) and indeterminate for one pride response (demand better treatment, BF_10_ = 1.65).

*Exploratory question: are there country-level differences in pride responses? And if so, are those differences patterned?* Yes, and yes. Whereas the five pride responses tended to be positively correlated with valuation both in the USA and in India, the distributions of these responses were somewhat different across the two countries (see Fig. 1). For example, ratings of valuation and ratings of pride responses tended to be higher in India than in the USA – the exception being pride feeling, which tended to be higher in the USA (Table S5). These differences in absolute levels of pride responses may be due to various causes, including, for example, the difference in mean age between our USA and India samples (10 years’ difference), considering that differences in age are associated with differences in levels of pride (see Orth et al., 2010, Fig. 1). Might these country-level differences be patterned? The advertisement–recalibration theory suggests that country-level differences in pride responses may stem from country-level differences in audience valuation. Consistent with this hypothesis, exploratory analyses indicated that country-level differences in pride responses were systematically correlated with country-level differences in audience valuation. For each scenario, and for each of the six target measures (valuation, pride feeling, communicate event, demand better treatment, invest in valued trait and pursue new challenges), we subtracted the mean ratings provided by American participants from the mean ratings provided by Indian participants. This resulted in 25 difference scores – one for each of the 25 scenarios – for each of the six measures. Difference scores of valuation were correlated positively and significantly with difference scores of pride feeling (*r* = 0.75, *P* = 0.00002), difference scores of communicate event (*r* = 0.61, *P* = 0.001), difference scores of invest in valued trait (*r* = 0.44, *P* = 0.027) and difference scores of pursue new challenges (*r* = 0.47, *P* = 0.019), and marginally with difference scores of demand better treatment (*r* = 0.35, *P* = 0.082). The more an act or trait was socially valued in others by Indian participants relative to American participants, the more that act or trait led to higher pride responses among Indian participants relative to American participants.

## Discussion

We argued that pride is an evolved emotion system that functions to exploit opportunities to become more highly valued and respected by others. We hypothesized that pride works by matching in intensity the various outputs it controls to the evaluations that other people make of one's achievements – thus balancing the competing demands of effectiveness and economy in its operation. To evaluate this hypothesis, we evaluated whether the intensities of various pride responses cohere in the general case where the inputs to the pride system are specified hypothetically and minimally and participants respond anticipatorily. The results support the hypothesis.

We observed internal coherence: the event-specific intensities of five pride responses – felt pride and the motivations to communicate the event, to demand better treatment, to invest in the valued trait and to pursue new challenges – in general correlated positively with one other. The present observations of internal coherence agree with some (but not all) of the previous affective science findings on response coherence.

Besides internal coherence, we predicted and observed two novel patterns of response coherence: external coherence and cross-cultural coherence. Regarding external coherence, the intensities of five pride responses in general correlated positively with the event-specific magnitude of social valuation expressed by audiences. We observed external (and internal) coherence within the USA and within India. Regarding cross-cultural coherence, the intensities of five pride responses in one culture in general correlated positively both with the intensities of the five pride responses and with the magnitude of audience valuation in the other culture. It is notable that multiple pride responses in one culture vary in sync with those same responses and with the magnitude of valuation conferred by audiences *in another culture*. However, this is expected if a system – pride – controls multiple responses and does so in Goldilocks fashion and if there are cross-cultural regularities in the information-processing structure and content of the system. In addition, the intensities of participants’ motivations to destroy evidence about their own achievements failed to correlate positively with the magnitude of audience valuation and with the intensities of the five pride responses. This suggests that the observed patterns of response coherence may not have been driven by similarities in valence, as the alternative theory of constructed emotion might predict. We note that destruction of evidence indicating positive social value of the self is one type of action that is arousing while lacking the adaptive functionality of promoting valuation from others. Therefore, while our tests against this particular alternative support the advertisement–recalibration theory, they do not rule out the broader class of alternative explanations involving arousal, or the even broader alternative theory of constructed emotion (which also involves valence and concepts as explanatory elements). Future research is needed to test against additional alternatives involving arousal, valence and culturally variable emotion concepts.

The coherence observed here does not seem obviously driven by cultural similarities between the USA and India in the concepts that people use to parse their core affect (another candidate predictor of cultural similarities in emotion according to the alternative theory of constructed emotion; Barrett, 2006b), as those concepts vary considerably, between individualist cultures and collectivist cultures (Eid & Diener, 2009; Mesquita, 2001; Neumann et al., 2009), and between the USA and India (Shweder, 2003). This point needs to be tempered, however. Data from India indicate substantial behavioural variation within a cultural group (Lamba & Mace, 2011) that is comparable in size with the variation observed between cultural groups (e.g. Henrich et al., 2005). Therefore, similarities and differences between (and within) populations need to be interpreted cautiously. There are similarities between the American and Indian participants who took part in the present study, after all (e.g. they are MTurk and internet users). So it could be that we effectively sampled a single culture. Likewise, the US–India similarities observed here may have been imparted by the particular concept of ‘pride’ in the English language and not by an evolved pride system – consider that emotion words have meanings that are more similar in language groups that are in closer geographic proximity (Jackson et al., 2019) and that our study was conducted in English in the USA and India. Alternatively, the USA–India similarities observed here may have been driven by the particular schemas with which citizens from industrial nations organize their experiences of pride, and not by an evolved pride system. These considerations may tip the interpretation of the results towards the alternative theory of constructed emotion. Note, however, that previous research has shown cross-cultural commonalities in the feeling of pride across populations with highly diverse subsistence modes (e.g. horticulture, pastoralism, wage labour) and speaking highly diverse languages (e.g. Mayangna, Moroccan Arabic, Igbo, Tuvan; Sznycer et al., 2018b). Thus, the USA–India similarities observed here might reflect the operation of a pride orchestrator after all.

Finally, exploratory analyses suggested that cultural differences in pride responses may stem from cultural differences in audience valuation.

In aggregate, the observed pattern of findings follows from and is consistent with the advertisement–recalibration theory of pride, although interpretative ambiguities remain, as noted above.

As stated above, some researchers have interpreted observations of low or inconsistent response coherence as indications that what is biologically real in emotion episodes is a host of neurocognitive systems (e.g. the systems generating core affect and concepts) and their interactions, *but not dedicated programmes with orchestrating functions* (Barrett, 2006a; Barrett & Russell, 2015). However, inconsistencies in response coherence need not be damning to the hypothesis that emotions are specialized neurocognitive systems if emotions solve adaptive problems by adaptively orchestrating responses (Tooby & Cosmides, 1990b; Cosmides & Tooby, 2000; Reisenzein, 2000; Scarantino, 2015). If emotions can match different inputs to different outputs facultatively and functionally, then coherent, stereotypical clusters of responses may be observed when the emotion architectures specify those clusters as the best-bet response given the present inputs (e.g. regarding pride, advertise the achievement when the audience is co-present) and may not be observed otherwise (e.g. do not advertise the achievement when the audience is not co-present). In addition, and more positively, the fact that pride responses can cohere across populations, and also externally, matching in magnitude the evaluative responses of audiences, suggests that it may be premature to conclude that pride, and perhaps other emotions, lacks dedicated orchestrating functions. We note that the ontological status of emotion is perhaps the foremost point of contention in the affective sciences (see, e.g. Adolphs & Anderson, 2018; Barrett, 2019; Barrett et al., 2018, 2019; Cowen & Keltner, 2017; Lindquist et al., 2013; Mobbs et al., 2019; Scarantino, 2015; Sznycer & Cohen, under review).

Further research is needed to know whether the patterns of coherence observed here are observed in other populations with diverse languages, ecologies and cultures; in other pride responses; and in the reactive mode of pride in response to achievements actually attained. Further research is also needed to know whether and the extent to which pride (and other emotions) can orchestrate their responses contingently as a function of varying inputs. Perhaps the inconsistencies in response coherence reported so far in the emotion literature are due to emotions orchestrating their outputs contingently – although, as noted above, inconsistencies in coherence may also be due to other causes.

In conclusion, these findings suggest that the emotion of pride works by precisely matching the various valuation-promoting outputs at its disposal to the degree to which other people would value the individual if the individual took a socially valued act or possessed a socially valued characteristic. This magnitude-matching feature bears the mark of natural selection, because this feature is improbably well suited to balance the competing demands of effectiveness and economy (Williams, 1966). More generally, these findings suggest that pride functions to promote the pursuit of socially valued courses of action and to facilitate the gains in social valuation that make those actions worth pursuing.

## Supplementary material

For supplementary material accompanying this paper visit https://doi.org/10.1017/ehs.2021.6. click here to view supplementary material

## Data access

Preregistration protocol: https://aspredicted.org/4k5wq.pdf. Study materials and dataset: https://osf.io/tr8fe/
