# Supplementary material for: How pride works
Source: Evol Hum Sci. 2021 Feb 1;3:e10. doi: 10.1017/ehs.2021.6 (PMC10427325; doi:10.1017/ehs.2021.6)
Supplement: Supplementary file 1 [file S2513843X21000062sup.zip › S2513843X21000062sup002.docx]

**Appendix**

**How pride works**

**Table S1**

*Condition prompts and anchors*

| *Condition* | *Prompt* | *Low anchor (1)* | *High anchor (7)* |
| --- | --- | --- | --- |
|  |  |  |  |
| Valuation | Next, we will ask you to imagine several hypothetical situations.  Imagine that the things described in the hypothetical situations are true of another person of your same sex and age.  Please indicate how positively you would view this person if those things were true of that person. | I'd view him not positively at all if this were true of him | I'd view him very positively if this were true of him |
|  |  |  |  |
| Pride feeling | Next, we will ask you to imagine several hypothetical situations.  Imagine that the things described in the hypothetical situations are true of you. Please indicate how much pride you would feel if those things were true of you. | No pride at all if this were true of me | A lot of pride if this were true of me |
|  |  |  |  |
| Communicate event | Next, we will ask you to imagine several hypothetical situations.  Imagine that the things described in the hypothetical situations are true of you. Please indicate how willing you would be to communicate to others that those things are true of you. | Not at all willing to communicate to others that this is true of me | Very much willing to communicate to others that this is true of me |
|  |  |  |  |
| Demand better treatment | Next, we will ask you to imagine several hypothetical situations.  Imagine that the things described in the hypothetical situations are true of you. Please indicate how willing you would be to demand others to treat you better because of those things. | Not at all willing to demand others to treat me better because of this | Very much willing to demand others to treat me better because of this |
|  |  |  |  |
| Invest in valued trait | Next, we will ask you to imagine several hypothetical situations.  Imagine that the things described in the hypothetical situations are true of you. Please indicate how willing you would be to invest (time, effort, etc.) so that those things continue to be true of you. | Not at all willing to invest for this to continue to be true of me | Very much willing to invest for this to continue to be true of me |
|  |  |  |  |
| Pursue new challenges | Next, we will ask you to imagine several hypothetical situations.  Imagine that the things described in the hypothetical situations are true of you. Please indicate how much, because of those things, you would be motivated to pursue new life challenges. | I’d not at all be motivated to pursue new life challenges if this were true of me | I’d very much be motivated to pursue new life challenges if this were true of me |
|  |  |  |  |
| Destroy evidence | Next, we will ask you to imagine several hypothetical situations.  Imagine that the things described in the hypothetical situations are true of you.  Please indicate how willing you would be to destroy evidence or clues that might tell others that those things are true of you. | Not at all willing to destroy evidence or clues that might tell others that this is true of me | Very much willing to destroy evidence or clues that might tell others that this is true of me |

Note: The male versions of the prompts are presented.

**Table S2**

*Ratings of valuation, pride feeling, communicate event, demand better treatment, invest in valued trait, pursue new challenges, and destroy evidence, by scenario: United States*

| # | Scenario | Valuation | Pride feeling | Communicate event | Demand better treatment | Invest in valued trait | Pursue new challenges | Destroy evidence |
| --- | --- | --- | --- | --- | --- | --- | --- | --- |
| 14 | You are generous with others. / He is generous with others. | 6.53 (0.90) | 6.17 (1.13) | 5.11 (1.86) | 3.17 (2.11) | 5.91 (0.98) | 5.15 (1.73) | 2.56 (1.80) |
| 18 | You are trustworthy. / He is trustworthy. | 6.50 (0.90) | 6.22 (0.87) | 6.06 (1.41) | 3.54 (2.08) | 6.23 (0.94) | 5.03 (1.67) | 1.50 (0.96) |
| 4 | You take very good care of your children. / He takes very good care of his children. | 6.41 (1.05) | 6.50 (0.70) | 5.86 (1.59) | 3.23 (2.10) | 6.46 (0.95) | 5.00 (1.62) | 1.47 (1.02) |
| 19 | When there is a conflict in your community, people ask you to mediate between the two sides. / When there is a conflict in his community, people ask him to mediate between the two sides. | 6.06 (0.98) | 5.14 (1.44) | 4.53 (2.02) | 3.34 (2.15) | 4.00 (1.64) | 5.24 (1.48) | 2.62 (1.56) |
| 25 | People think that you are the bravest man of your community. / People think that he is the bravest man of his community. | 6.03 (1.11) | 5.81 (1.06) | 3.56 (1.86) | 2.86 (2.13) | 4.20 (1.95) | 5.33 (1.53) | 2.53 (1.67) |
| 5 | You can support your children economically. / He can support his children economically. | 6.00 (1.18) | 6.31 (0.79) | 5.31 (1.88) | 3.20 (2.15) | 6.17 (1.34) | 5.24 (1.56) | 1.74 (1.14) |
| 8 | You are very smart. / He is very smart. | 5.97 (1.19) | 6.17 (1.03) | 4.58 (1.79) | 3.00 (2.14) | 5.91 (1.27) | 6.06 (1.25) | 2.18 (1.80) |
| 24 | You finished first in a marathon. / He finished first in a marathon. | 5.94 (1.01) | 5.89 (1.63) | 4.94 (2.40) | 2.80 (1.92) | 4.14 (2.05) | 5.48 (1.25) | 2.09 (1.44) |
| 16 | You have many unique skills. / He has many unique skills. | 5.76 (1.33) | 6.00 (1.35) | 5.58 (1.08) | 3.26 (2.15) | 5.74 (1.17) | 6.21 (0.82) | 2.09 (1.50) |
| 22 | People love your sense of humor. / People love his sense of humor. | 5.76 (1.28) | 5.94 (0.95) | 5.31 (1.51) | 2.60 (1.87) | 5.77 (1.00) | 5.09 (1.38) | 1.97 (1.29) |
| 2 | You host your extended family for a holiday meal; they think it’s the best meal they’ve ever had. / He hosts his extended family for a holiday meal; they think it’s the best meal they’ve ever had. | 5.74 (1.11) | 5.89 (1.17) | 4.92 (1.89) | 2.83 (1.87) | 5.40 (1.58) | 5.06 (1.50) | 1.76 (1.16) |
| 17 | You have a lot of good friends. / He has a lot of good friends. | 5.59 (1.33) | 5.86 (1.13) | 5.14 (2.03) | 2.63 (2.06) | 5.26 (1.72) | 4.88 (1.73) | 2.00 (1.33) |
| 10 | You have good table manners. / He has good table manners. | 5.56 (1.21) | 4.72 (1.30) | 4.81 (2.07) | 2.74 (1.96) | 5.57 (1.48) | 4.18 (1.81) | 1.71 (1.17) |
| 15 | You are ambitious. / He is ambitious. | 5.32 (1.47) | 5.75 (1.42) | 5.19 (1.83) | 2.94 (2.04) | 5.46 (1.72) | 6.21 (0.82) | 2.41 (1.69) |
| 9 | You have more years of education than those around you. / He has more years of education than those around him. | 5.09 (1.36) | 5.03 (1.46) | 3.33 (1.84) | 2.57 (1.96) | 4.89 (1.81) | 5.61 (1.41) | 2.29 (1.64) |
| 12 | You are physically attractive. / He is physically attractive. | 4.94 (1.30) | 5.67 (1.15) | 3.78 (2.09) | 2.54 (2.12) | 5.57 (1.22) | 5.30 (1.36) | 2.35 (1.65) |
| 3 | Your children are healthier and taller than average for their age.  / His children are healthier and taller than average for their age. | 4.79 (1.45) | 5.11 (1.30) | 4.31 (1.88) | 1.97 (1.62) | 5.57 (1.38) | 4.21 (2.00) | 1.82 (1.17) |
| 6 | You are playing a throwing game with your friends. All your throws hit the target. / He is playing a throwing game with his friends. All his throws hit the target. | 4.79 (1.41) | 5.17 (1.40) | 5.11 (1.77) | 2.46 (1.92) | 4.31 (1.71) | 4.48 (1.79) | 1.88 (1.32) |
| 1 | You look ten years younger than you are. / He looks ten years younger than he is. | 4.71 (1.57) | 5.36 (1.33) | 3.86 (2.11) | 2.23 (1.91) | 5.29 (1.76) | 4.94 (1.50) | 2.29 (1.77) |
| 13 | You are wealthy. / He is wealthy. | 4.68 (1.51) | 5.19 (1.43) | 2.67 (1.66) | 2.66 (2.10) | 5.80 (1.30) | 5.67 (1.51) | 3.97 (1.90) |
| 20 | Your wife is the most attractive woman in your community. / His wife is the most attractive woman in his community. | 4.41 (1.28) | 5.22 (1.33) | 3.81 (2.18) | 2.49 (2.09) | 4.60 (1.99) | 4.33 (1.81) | 2.18 (1.64) |
| 7 | You come from a wealthy family with high status and many connections. / He comes from a wealthy family with high status and many connections. | 4.24 (1.50) | 4.72 (1.72) | 2.64 (1.79) | 2.57 (1.97) | 4.54 (1.90) | 5.64 (1.62) | 4.00 (2.00) |
| 21 | You are the tallest in your circle of friends. / He is the tallest in his circle of friends. | 4.18 (1.34) | 3.61 (1.78) | 4.19 (2.20) | 2.06 (1.92) | 2.51 (1.67) | 3.85 (2.00) | 2.09 (1.50) |
| 23 | An acquaintance of yours had been bullying you for a while. At some point you got tired of it, and you beat him very badly. You were never bullied again. / An acquaintance of his had been bullying him for a while. At some point he got tired of it, and he beat him very badly. He was never bullied again. | 3.68 (1.75) | 3.89 (2.05) | 3.50 (2.22) | 3.14 (1.96) | 3.63 (2.13) | 3.85 (2.02) | 4.03 (2.14) |
| 11 | You get into a fight in front of everybody, and you completely dominate your opponent with punch after punch, until your opponent is knocked out. / He gets into a fight in front of everybody, and he completely dominates his opponent with punch after punch, until his opponent is knocked out. | 2.53 (1.60) | 3.53 (2.16) | 2.58 (1.98) | 2.69 (2.01) | 2.74 (1.99) | 3.64 (1.93) | 4.85 (2.12) |

*Note*. Displayed are means, with standard deviations in parentheses. *N*s: valuation: 34; pride feeling: 36; communicate event: 36; demand better treatment: 35; invest in valued trait: 35; pursue new challenges: 33; destroy evidence: 34. The male versions of the pride feeling/communicate event/demand better treatment/invest in valued trait/pursue new challenges/destroy evidence scenarios and the valuation scenarios are presented before and after the slash, respectively. Scenarios are displayed from highest to lowest mean valuation scores.

**Table S3**

*Ratings of valuation, pride feeling, communicate event, demand better treatment, invest in valued trait, pursue new challenges, and destroy evidence, by scenario: India*

| # | Scenario | Valuation | Pride feeling | Communicate event | Demand better treatment | Invest in valued trait | Pursue new challenges | Destroy evidence |
| --- | --- | --- | --- | --- | --- | --- | --- | --- |
| 18 | You are trustworthy. / He is trustworthy. | 5.96 (1.06) | 5.77 (1.48) | 4.96 (1.80) | 5.42 (1.35) | 5.95 (0.97) | 5.52 (1.20) | 4.93 (1.52) |
| 10 | You have good table manners. / He has good table manners. | 5.92 (0.95) | 5.50 (1.30) | 5.42 (1.63) | 5.26 (1.56) | 5.52 (1.29) | 5.43 (1.08) | 4.93 (1.47) |
| 14 | You are generous with others. / He is generous with others. | 5.92 (0.91) | 5.35 (1.72) | 5.19 (1.70) | 5.26 (1.48) | 5.48 (1.08) | 5.35 (1.07) | 5.07 (1.47) |
| 4 | You take very good care of your children. / He takes very good care of his children. | 5.88 (0.93) | 5.73 (1.25) | 5.27 (1.71) | 5.26 (1.56) | 5.48 (1.17) | 5.43 (1.08) | 4.93 (1.62) |
| 17 | You have a lot of good friends. / He has a lot of good friends. | 5.84 (1.03) | 5.46 (1.42) | 5.62 (1.24) | 5.53 (1.26) | 5.90 (0.62) | 5.43 (1.38) | 5.04 (1.68) |
| 5 | You can support your children economically. / He can support his children economically. | 5.76 (1.01) | 5.50 (1.53) | 5.42 (1.65) | 5.16 (1.68) | 5.67 (1.20) | 5.52 (1.16) | 5.04 (1.74) |
| 8 | You are very smart. / He is very smart. | 5.76 (1.13) | 5.27 (1.73) | 5.35 (1.50) | 5.26 (1.56) | 5.67 (1.11) | 5.52 (1.12) | 5.11 (1.45) |
| 15 | You are ambitious. / He is ambitious. | 5.68 (0.85) | 5.23 (1.63) | 5.23 (1.18) | 5.26 (1.66) | 5.67 (1.02) | 5.78 (0.90) | 5.19 (1.21) |
| 16 | You have many unique skills. / He has many unique skills. | 5.64 (1.15) | 5.62 (1.44) | 4.88 (1.63) | 5.26 (1.19) | 5.43 (0.93) | 5.39 (1.16) | 5.04 (1.53) |
| 22 | People love your sense of humor. / People love his sense of humor. | 5.64 (1.08) | 5.46 (1.50) | 5.31 (1.57) | 5.37 (1.61) | 5.48 (1.03) | 5.43 (1.12) | 5.30 (0.99) |
| 1 | You look ten years younger than you are. / He looks ten years younger than he is. | 5.52 (1.00) | 5.27 (1.69) | 5.00 (1.81) | 5.00 (1.56) | 5.10 (1.41) | 5.87 (0.81) | 5.22 (1.15) |
| 9 | You have more years of education than those around you. / He has more years of education than those around him. | 5.48 (1.33) | 5.46 (1.27) | 4.96 (1.64) | 4.84 (1.95) | 5.29 (1.23) | 5.39 (1.08) | 4.74 (1.40) |
| 19 | When there is a conflict in your community, people ask you to mediate between the two sides. / When there is a conflict in his community, people ask him to mediate between the two sides. | 5.48 (1.29) | 4.92 (1.55) | 4.69 (1.87) | 5.42 (1.35) | 5.43 (1.21) | 4.96 (1.11) | 5.11 (1.37) |
| 12 | You are physically attractive. / He is physically attractive. | 5.44 (1.08) | 5.35 (1.65) | 5.08 (1.57) | 4.63 (1.67) | 5.95 (0.74) | 5.17 (1.56) | 5.37 (1.11) |
| 2 | You host your extended family for a holiday meal; they think it’s the best meal they’ve ever had. / He hosts his extended family for a holiday meal; they think it’s the best meal they’ve ever had. | 5.36 (1.29) | 5.58 (1.24) | 4.88 (1.73) | 5.21 (1.62) | 5.38 (1.20) | 5.48 (1.24) | 5.26 (1.29) |
| 7 | You come from a wealthy family with high status and many connections. / He comes from a wealthy family with high status and many connections. | 5.32 (1.28) | 5.62 (1.06) | 5.19 (1.67) | 5.16 (1.71) | 5.14 (1.31) | 5.70 (0.93) | 5.19 (1.21) |
| 3 | Your children are healthier and taller than average for their age.  / His children are healthier and taller than average for their age. | 5.28 (1.06) | 5.50 (1.24) | 4.77 (1.92) | 5.00 (1.70) | 5.43 (1.16) | 5.30 (1.26) | 4.63 (1.57) |
| 20 | Your wife is the most attractive woman in your community. / His wife is the most attractive woman in his community. | 5.24 (1.36) | 5.19 (1.44) | 5.00 (2.06) | 5.05 (1.72) | 5.62 (0.80) | 5.30 (1.36) | 5.33 (1.04) |
| 24 | You finished first in a marathon. / He finished first in a marathon. | 5.24 (1.45) | 5.62 (1.50) | 5.15 (1.57) | 5.21 (1.65) | 4.95 (1.69) | 5.26 (1.51) | 5.00 (1.36) |
| 6 | You are playing a throwing game with your friends. All your throws hit the target. / He is playing a throwing game with his friends. All his throws hit the target. | 5.20 (1.63) | 5.42 (1.58) | 5.08 (1.62) | 5.47 (1.39) | 5.19 (1.17) | 5.30 (1.11) | 5.52 (0.98) |
| 13 | You are wealthy. / He is wealthy. | 5.16 (1.21) | 5.19 (1.44) | 4.58 (1.84) | 5.16 (1.26) | 5.10 (1.26) | 5.26 (1.45) | 5.33 (1.14) |
| 25 | People think that you are the bravest man of your community. / People think that he is the bravest man of his community. | 5.16 (1.25) | 5.23 (1.73) | 5.19 (1.74) | 5.42 (1.30) | 5.62 (1.24) | 5.48 (1.12) | 4.81 (1.27) |
| 21 | You are the tallest in your circle of friends. / He is the tallest in his circle of friends. | 4.88 (1.48) | 4.69 (1.46) | 4.46 (1.92) | 4.68 (1.95) | 4.76 (1.37) | 4.96 (1.52) | 5.04 (1.43) |
| 23 | An acquaintance of yours had been bullying you for a while. At some point you got tired of it, and you beat him very badly. You were never bullied again. / An acquaintance of his had been bullying him for a while. At some point he got tired of it, and he beat him very badly. He was never bullied again. | 4.64 (1.47) | 4.35 (1.79) | 4.88 (1.53) | 5.26 (1.56) | 5.19 (0.98) | 4.91 (1.53) | 4.85 (1.41) |
| 11 | You get into a fight in front of everybody, and you completely dominate your opponent with punch after punch, until your opponent is knocked out. / He gets into a fight in front of everybody, and he completely dominates his opponent with punch after punch, until his opponent is knocked out. | 4.52 (1.71) | 4.46 (1.63) | 4.58 (1.79) | 5.11 (1.70) | 5.19 (1.36) | 4.78 (1.20) | 4.96 (1.34) |

*Note*. Displayed are means, with standard deviations in parentheses. *N*s: valuation: 25; pride feeling: 26; communicate event: 26; demand better treatment: 19; invest in valued trait: 21; pursue new challenges: 23; destroy evidence: 27. The male versions of the pride feeling/communicate event/demand better treatment/invest in valued trait/pursue new challenges/destroy evidence scenarios and the valuation scenarios are presented before and after the slash, respectively. Scenarios are displayed from highest to lowest mean valuation scores.

**Table S4**

Correlations between conditions, within- and between-countries.

|  | (1) | (2) | (3) | (4) | (5) | (6) | (7) | (8) | (9) | (10) | (11) | (12) | (13) | (14) |
| --- | --- | --- | --- | --- | --- | --- | --- | --- | --- | --- | --- | --- | --- | --- |
| (1) US_Valuation |  |  |  |  |  |  |  |  |  |  |  |  |  |  |
| (2) US_Pride | .87 |  |  |  |  |  |  | .78 |  |  |  |  |  |  |
| (3) US_Communicate | .75 | .67 |  |  |  |  |  | .70 | .54 |  |  |  |  |  |
| (4) US_Demand | .55 | .46 | .46 |  |  |  |  | .40 | .13 | .23 |  |  |  |  |
| (5) US_Invest | .67 | .82 | .53 | .34 |  |  |  | .83 | .74 | .53 | .21 |  |  |  |
| (6) US_Pursue | .55 | .66 | .20 | .38 | .53 |  |  | .51 | .52 | .32 | .16 | .26 |  |  |
| (7) US_Destroy | −.71 | −.62 | −.80 | −.05 | −.51 | −.15 |  | −.67 | −.65 | −.42 | −.09 | −.38 | −.41 |  |
| (8) IN_Valuation | .82 |  |  |  |  |  |  |  |  |  |  |  |  |  |
| (9) IN_Pride | .68 | .76 |  |  |  |  |  | .74 |  |  |  |  |  |  |
| (10) IN_Communicate | .52 | .60 | .45 |  |  |  |  | .67 | .53 |  |  |  |  |  |
| (11) IN_Demand | .44 | .38 | .44 | .55 |  |  |  | .31 | .20 | .42 |  |  |  |  |
| (12) IN_Invest | .48 | .59 | .41 | .41 | .59 |  |  | .61 | .36 | .55 | .28 |  |  |  |
| (13) IN_Pursue | .45 | .61 | .30 | .02 | .64 | .57 |  | .64 | .69 | .59 | .20 | .30 |  |  |
| (14) IN_Destroy | −.07 | .13 | .00 | −.12 | .07 | .19 | .03 | .02 | .05 | .04 | .03 | −.01 | .14 |  |

*Note*: Coefficients are Pearson's *r*s. N on which the correlations are based = number of scenarios = 25. Shaded cells: within-country correlations; non-shaded cells: between-country correlations. US: United States; IN: India. Ratings of valuation, pride feeling, communicate event, demand better treatment, invest in valued trait, pursue new challenges, and destroy evidence were given by different participants. All |*r*s| ≥ .39 have *P*s < or << .05.

**Table S5**

*Country-level differences in valuation, pride feeling, communicate event, demand better treatment, invest in valued trait, and pursue new challenges, by scenario*

| # | Scenario | Valuation | Pride feeling | Communicate event | Demand better treatment | Invest in valued trait | Pursue new challenges |
| --- | --- | --- | --- | --- | --- | --- | --- |
| 1 | You look ten years younger than you are. / He looks ten years younger than he is. | **.31** | −.03 | **.28** | **.60** | −.06 | **.38** |
| 2 | You host your extended family for a holiday meal; they think it’s the best meal they’ve ever had. / He hosts his extended family for a holiday meal; they think it’s the best meal they’ve ever had. | −.16 | −.13 | −.01 | **.54** | −.01 | .15 |
| 3 | Your children are healthier and taller than average for their age.  / His children are healthier and taller than average for their age. | .18 | .15 | .12 | **.67** | −.05 | **.32** |
| 4 | You take very good care of your children. / He takes very good care of his children. | **−.26** | **−.43** | −.18 | **.51** | **−.42** | .15 |
| 5 | You can support your children economically. / He can support his children economically. | −.11 | **−.39** | .03 | **.48** | −.19 | .10 |
| 6 | You are playing a throwing game with your friends. All your throws hit the target. / He is playing a throwing game with his friends. All his throws hit the target. | .13 | .09 | −.01 | **.69** | **.27** | **.28** |
| 7 | You come from a wealthy family with high status and many connections. / He comes from a wealthy family with high status and many connections. | **.36** | **.31** | **.59** | **.55** | .17 | .02 |
| 8 | You are very smart. / He is very smart. | −.09 | **−.36** | .22 | **.54** | −.10 | −.22 |
| 9 | You have more years of education than those around you. / He has more years of education than those around him. | .14 | .15 | **.42** | **.49** | .12 | −.08 |
| 10 | You have good table manners. / He has good table manners. | .16 | **.29** | .16 | **.61** | −.02 | **.41** |
| 11 | You get into a fight in front of everybody, and you completely dominate your opponent with punch after punch, until your opponent is knocked out. / He gets into a fight in front of everybody, and he completely dominates his opponent with punch after punch, until his opponent is knocked out. | **.52** | .24 | **.47** | **.53** | **.60** | **.35** |
| 12 | You are physically attractive. / He is physically attractive. | .20 | −.12 | **.34** | **.46** | .19 | −.04 |
| 13 | You are wealthy. / He is wealthy. | .17 | .00 | **.48** | **.61** | −.26 | −.14 |
| 14 | You are generous with others. / He is generous with others. | **−.32** | **−.32** | .02 | **.52** | −.21 | .07 |
| 15 | You are ambitious. / He is ambitious. | .16 | −.17 | .01 | **.56** | .08 | −.24 |
| 16 | You have many unique skills. / He has many unique skills. | −.05 | −.14 | **−.25** | **.52** | −.14 | **−.43** |
| 17 | You have a lot of good friends. / He has a lot of good friends. | .11 | −.16 | .15 | **.67** | **.28** | .17 |
| 18 | You are trustworthy. / He is trustworthy. | **−.27** | −.22 | **−.33** | **.49** | −.14 | .16 |
| 19 | When there is a conflict in your community, people ask you to mediate between the two sides. / When there is a conflict in his community, people ask him to mediate between the two sides. | −.25 | −.07 | .04 | **.52** | **.43** | −.11 |
| 20 | Your wife is the most attractive woman in your community. / His wife is the most attractive woman in his community. | **.30** | −.01 | **.27** | **.54** | **.36** | **.28** |
| 21 | You are the tallest in your circle of friends. / He is the tallest in his circle of friends. | .25 | **.31** | .06 | **.55** | **.62** | **.31** |
| 22 | People love your sense of humor. / People love his sense of humor. | −.05 | −.22 | .00 | **.66** | −.14 | .13 |
| 23 | An acquaintance of yours had been bullying you for a while. At some point you got tired of it, and you beat him very badly. You were never bullied again. / An acquaintance of his had been bullying him for a while. At some point he got tired of it, and he beat him very badly. He was never bullied again. | **.28** | .12 | **.35** | **.49** | **.46** | **.29** |
| 24 | You finished first in a marathon. / He finished first in a marathon. | **−.28** | −.09 | .05 | **.60** | .20 | −.08 |
| 25 | People think that you are the bravest man of your community. / People think that he is the bravest man of his community. | **−.35** | −.24 | **.41** | **.61** | **.41** | .05 |
| Across all scenarios | | .12 | −.05 | **.28** | **.65** | .24 | .19 |

*Note*. Displayed are the effect sizes (*r*) corresponding to the country-level differences. Positive values: India mean > United States mean; negative values: India mean < United States mean; bolded values: *p*-value of the difference < or << .05; non-bolded values: p-value of the difference ≥ .05.
